# Development of Nuclear DNA Markers for Applications in Genetic Diversity Study of Oil Palm-Pollinating Weevil Populations

**DOI:** 10.3390/insects14020157

**Published:** 2023-02-03

**Authors:** Fairuz Farhana Mohd Rodzik, Nurshazwani Amalina Sudirman, Chee-Keng Teh, Ai-Ling Ong, Huey-Ying Heng, Salmah Yaakop, Norfarhan Mohd-Assaad, Meilina Ong-Abdullah, Nabeel Ata, Samsudin Amit, Burhanuddin Saragih, David Ross Appleton, Harikrishna Kulaveerasingam

**Affiliations:** 1 Sime Darby Plantation R&D Centre, Serdang 43400, Selangor, Malaysia; 2 Centre for Insect Systematics, Department of Biological Science and Biotechnology, Faculty of Science and Technology, Universiti Kebangsaan Malaysia (UKM), Bangi 43600, Selangor, Malaysia; 3 Department of Applied Physics, Faculty of Science and Technology, Universiti Kebangsaan Malaysia (UKM), Bangi 43600, Selangor, Malaysia; 4 Institute of Systems Biology (INBIOSIS), Universiti Kebangsaan Malaysia, Bangi 43600, Selangor, Malaysia; 5 Advanced Biotechnology and Breeding Centre, Malaysian Palm Oil Board (MPOB), 6 Persiaran Institusi, Bandar Baru Bangi, Kajang 43000, Selangor, Malaysia; 6 Minamas Research Centre Pekanbaru, Jalan Baru Bakal, Tualang Timur, Kecamatan Tualang, Kabupaten Siak, Perawang 28772, Provinsi Riau, Indonesia

**Keywords:** inbreeding depression, simple sequence repeats (SSR), single nucleotide polymorphisms (SNP), molecular markers, restriction-site associated DNA (RAD), pollinating agent

## Abstract

**Simple Summary:**

The main pollinator for the African oil palm, *Elaeis guineensis*, in Malaysia and Indonesia is *Elaeidobius kamerunicus*. The weevil species is not native to these countries, but it was introduced from Cameroon, West Africa, in 1981 to improve pollination efficiency, thus improving bunch formation and yield. Forty years after the weevil introduction, recent reductions in bunch yield reported in Malaysia caused by poor bunch formation could be associated with a decrease in pollination efficiency due to the founder effect. Several genetic diversity studies of weevil populations based on morphological and mitochondrial markers have been carried out; however, the studies did not provide sufficient evidence for explaining the genetic variation, particularly at the intra-species level. This study aims to develop a set of robust *E. kamerunicus*-specific nuclear DNA markers to directly assess the genetic diversity of weevil populations. The marker development lays the foundation for future applications by extending the survey into larger areas where the oil palm is cultivated to obtain a more comprehensive understanding of the genetic diversity and inbreeding occurrence status of *E. kamerunicus* in these introduced regions. This could facilitate sustainable genetic monitoring and conservation planning of *E. kamerunicus*, especially in non-native oil palm-growing countries.

**Abstract:**

The oil palm-pollinating weevil (*Elaeidobius kamerunicus* Faust) was introduced from Cameroon, West Africa, to Malaysia in 1981, and subsequently, to other oil palm-growing countries as well. This study aims to develop a set of robust *E. kamerunicus*-specific nuclear DNA markers to directly assess the genetic diversity of the weevil populations. A total of 19,148 SNP and 223,200 SSR were discovered from 48 weevils representing three origins (Peninsular Malaysia, Sabah, and Riau) using RAD tag sequencing. Subsequent filtering steps further reduced these to 1000 SNP and 120 SSR. The selected 220 SNP exhibited a polymorphism information content (PIC) of 0.2387 (±0.1280), and 8 SSR had the PIC of 0.5084 (±0.1928). These markers were found to show sufficient polymorphism, making it possible to assign 180 weevils into three major clusters from Ghana, Cameroon, and Southeast Asia (mainly in Malaysia and Indonesia). These DNA markers successfully confirmed the Cameroon origin of the Southeast Asian cluster. However, the presence of null alleles in the SSR markers, due to limited flexibility of the probe design on the short RAD tags, led to an underestimation of heterozygosity within the populations. Hence, the developed SNP markers turned out to be more efficient than the SSR markers in the genetic diversity assessment of the *E. kamerunicus* populations. The genetic information provides useful insight into developing guidelines for the genetic monitoring and conservation planning of *E. kamerunicus*.

## 1. Introduction

Oil palm, *Elaeis guineensis* Jacq., originated from West Africa. The earliest record of its introduction to Southeast Asia is found regarding the planting of four *dura* palms in Bogor Botanical Garden, Indonesia, in 1848 [1]. Currently, descendants of these palms have become the major planting material of Southeast Asia and Oceania [2], supplying about 34.3% of the total palm oil trade [3]. The oil crop is monoecious, producing male and female inflorescences in an alternating cycle to promote cross pollination among palms for fruit bunch development, mainly assisted by weevils under genus *Elaeidobius* in West Africa [4]. However, the native pollinator was unavailable in Southeast Asia when the oil palm was first planted in the region. A lower than 60% fruit-to-bunch ratio indicates inefficient pollination, which can cause a significant yield loss of fresh fruit bunches (FFB) [5]. In the late 1970s, *Thrips hawaiiensis* was identified in Malaysia as an alternative pollinator to the native weevil, but the species was far less efficient and usually absent from young plantings [4]. Hence, the commercial plantings were mainly hand pollinated, which was extremely costly and laborious [6]. The oil palm industry was left with no other option but to consider introducing the native pollinator from Cameroon to Malaysia [7].

The species *Elaeidobius kamerunicus* Faust, 1898 (Coleoptera: Curculionidae) was chosen after the species was studied to be the most predominant oil palm pollinating weevil in Cameroon. The species is also well adapted to both wet and hot seasons, and has a high pollen carrying capacity among the other *Elaeidobius* species [7]. In addition, host-specificity testing found that the species was unable to complete its life cycle on any plant species in the country other than the oil palm; thus, it was deemed safe to be introduced [8]. *Elaeidobius kamerunicus* is holometabolous, completing its development on post anthesis oil palm male inflorescence [9,10]. Adult female oviposit eggs on the male oil palm inflorescence, and the larva emerge and undergo perfect metamorphosis. The adult (weevil), attracted to the strong aniseed smell produced by male flowers at anthesis, visits the male flowers as a food source (nectar) and oviposits its eggs. Pollen grains are attached to the adult weevils’ body, mostly on the elytra, but some on the ventral part of the thorax and abdomen and under the elytra [4]. The weevil visit the female flowers by deception, as they are attracted to the same aniseed smell given off by male flower at the receptive stage. This results in the transference of pollens from the male to the female flowers (pollination) [11].

In 1981, the *E. kamerunicus* species in the form of pupae was imported from Plantations Pamol du Cameroon, Lobe Estate, Cameroon, to Malaysia [12]. A total of 3000 adult weevils were reared and first released into two plantations in Malaysia in 1981; the introduction was a huge success. The rapid establishment of weevils throughout the plantations led to significant improvements in fruit set and oil yield [6,12,13]. Hand pollination was then terminated, with an estimated saving of MYR 50 million per year during the 1980s [6]. The release of weevil species extended to Indonesia, Papua New Guinea, Thailand, and Colombia revolutionized the entire palm oil industry [2,14].

A declining trend of FFB, however, has occasionally been reported in Malaysia since the 1990s. Low rainfall and over-pruning, causing a reduction in the formation of female inflorescences, could be contributing factors to this trend. In addition, the descendant population of introduced *E. kamerunicus* probably suffered a bottleneck effect that weakened their adaptability to local climate change, making them vulnerable to parasitic nematode infection [15,16,17,18]. This may directly reduce the pollination ability of the insect. Many studies on the genetic diversity of *E. kamerunicus* populations were conducted to determine the presence of a bottleneck effect, but multivariate analysis based on 15 morphological characteristics of *E. kamerunicus* mainly explained the variation between male and female insects, rather than between geographical regions [19]. Due to strong influences from the environment, some of these morphologic traits could mask the genetic variations [20]; therefore, the founder effect still remains uncertain.

Hence, geneticists started adopting molecular markers to directly measure the genetic diversity of this important weevil population. Mitochondrial marker *COI*, *COII* and a nuclear gene fragment of arginine kinase (*AK*) were used in estimating the phylogeography of the *E. kamerunicus* populations [21]. The South American and Asian populations were found to have originated from West and Central Tropical Africa [21]. However, mitochondrial markers alone may not be sufficient to detect genetic polymorphism for an intra-species population; hence, the use of a nuclear marker was suggested [22]. Nuclear markers, which include single nucleotide polymorphism (SNP) and simple sequence repeat (SSR) markers, are able to detect allele heterozygosity information, hence more useful than mitochondrial markers in insects’ genetic diversity studies [23]. Simple sequence repeats (SSR) have the advantage of being multi-allelic (having a higher marker polymorphism than SNP). In addition, SSR can be genotyped using a standard PCR-based method, thus possessing a simpler start-up requirement. On the other hand, SNP is bi-allelic, which means that it contains less genetic information per locus than SSR markers, as presented in [24]. Nevertheless, it has the capacity to be used in a high-throughput system to genotype a high number of SNP markers with a large sample size [25].

We hypothesize that the designed novel DNA markers will be effective to measure the inbreeding effect among the Malaysia populations, as well as to estimate the genetic diversity of the other introduced region (Indonesia), and to contrast with this effect with those of the origin countries (Cameroon and Ghana). Hence, the aim of this study was to develop two robust *E. kamerunicus*-specific nuclear marker systems, i.e., single nucleotide polymorphisms (SNP) and simple sequence repeats (SSR), using RAD (restriction site associated DNA) tag sequencing, and then to assess the effectiveness and applicability of the two marker sets to capture the expected genetic patterns in the population.

## 2. Materials and Methods

### 2.1. Sample Collection and Genomic DNA Extraction

A total of 48 individual weevils originating from Malaysia (40 individual weevils representing northern, central, southern Peninsular Malaysia, as well as 5 individual weevils from Sabah) and Indonesia (3 individual weevils from Riau) were collected for marker discovery and development. Tissues from the head and membranous part of the hind wings of each weevil were sampled for DNA extraction using Analytik Jena^TM^ Tick DNA kit (Analytik Jena, Berlin, Germany), according to the manufacturer’s protocol. This was to ensure good quality DNA for the RAD tag sequencing and to minimize the risk of cross-contamination from other organisms, particularly nematodes, which are usually present in the abdominal segment of weevils. Subsequently, the evaluation of marker applicability for genetic diversity studies was performed, based on a larger set of 180 weevils representing two native populations (from Cameroon and Ghana) and 10 introduced populations in Malaysia and Indonesia (Table 1); each population consists of 15 individuals. The genomic DNA was extracted using Sbeadex™ Mini kit (LGC Genomics, Hoddesdon, UK) in an oKtopure™ (LGC Genomics, Hoddesdon, UK). Standard protocols, with the modification of an additional washing step using 80% ethanol (150 µL) and 25 µL of Proteinase-K, were performed. The quality of the extracted DNA was quantified using a Microplate reader (ThermoFisher Scientific, Massachusetts, USA).

### 2.2. RAD Tag Sequencing and Sequence Alignment

The genomic DNA of each individual weevil was digested using the *Msll* restriction enzyme. Digested DNA fragments were subjected to 150 bases paired-end sequencing using Illumina NextSeq500 V2 platform (Illumina Inc., California, USA). Standard pre-processing steps were taken after the de-multiplexing of the library groups using Illumina bcl2fastq 2.17.1.14 software (Illumina Inc., California, USA). Raw sequences per sample were obtained by the de-multiplexing of library groups into samples, according to their inline barcodes (with a maximum of 2 mismatches or Ns) and verification of the restriction site. All reads were processed by an adaptor clipper, and reads with a final length shorter than 20 bases were discarded as well. The next step was quality trimming by removing reads containing Ns and at the 3′-end to get the average Phred quality score (Q > 20) within a window size of 10 bases. Processed sequence reads were pooled and clustered using the CD-HIT-EST program at 95% similarity cut-off [26]. The alignment of the subsampled quality-trimmed reads against the cluster sequences was performed using BWA version 0.7.12 [27]. Variant discovery and genotyping were then performed using Freebayes v1.0.2-16 [28] by following the parameters set to minimum base quality of 10, a minimum coverage of 5, a mismatch base quality threshold of 10, a minimum supporting allele qsum of 10, and a read mismatch limit of 3. These combined variants, scored in a variant call format (vcf) file, were re-filtered for the read count, ensuring that each locus showed at least 8 reads and a minor allele frequency >5%. Lastly, the genotypes must be observed in at least 32 out of 48 samples.

### 2.3. SNP Discovery and Genotyping

An in-house script was used to check the availability of minimal 75 bp upstream and downstream flanking sequences of each SNP. The fastacmd program [29] under BLAST [30] was used to extract the SNP markers with adequate flanking sequences. Remapping of these markers on the tag sequence pool was performed using BLASTN with an e-value threshold of 1e-05 and 95% identity over a minimum of 140 bp to ensure high specificity to the targeted SNP markers. In addition, unique markers were then mapped against the protein (NR) database and the bacteria complete genome from the NCBI database, using BLASTN with a maximum e-value of 1e-05. The list of bacteria can be found at ftp://ftp.ncbi.nlm.nih.gov/genomes/refseq/bacteria/assembly_summary.txt (accessed on 26 January 2018). This step was performed to remove possible contaminants from weevil-specific sequences. In addition, as *E. kamerunicus* belongs to the order Coleoptera, those identified markers that were mapped to genomes of beetle species (*Aethina tumida, Agriotes lineatus, Anoplophora glabripennis, Biphyllus lunatus, Carabus granulatus, Dascillus cervinus, Dendroctonus ponderosae, Julodis onopordi, Leptinotarsa decemlineata, Onthophagus taurus, Scarabaeus laticollis,* and *Tribolium castaneum*) in the NR database were selected for this study. Additionally, those unmapped markers with a >90% call rate calculated in the PLINK program [31] were selected to eventually make up the 1000 SNP markers. To validate these candidate SNP markers, 75 bp upstream and downstream from an SNP were probed to genotype a total of 180 weevil samples using SeqSNP^TM^ Genotyping by Sequencing (LGC Genomics, Berlin, Germany. Only SNP with a >95% call rate were retained for subsequent analysis [32].

### 2.4. SSR Discovery and Genotyping

FullSSR suite v1.0 [33] was adopted to identify SSR. Unique contigs that contained the SSR motifs were confirmed by aligning all-against-all BLASTN at the e-value threshold of 1e-25 to the cluster sequences. Only those assured contigs harboring SSR motifs with > 8X coverage in BAM files were then imported into the Primer Premier 6 program (PREMIER Biosoft, US) for primer design. The design complied with a primer length ranging from 18 bases to 23 bases, target PCR products ranging from 100 bp to 280 bp, and a melting temperature (T_m_) ranging from 54 °C to 60 °C; the maximum T_m_ difference between the forward and the reverse primer was set at 0.5 °C. A total of 120 primer pairs with an M13-tailed forward primer for subsequent amplicon dye-labeling [34] were synthesized (IDT, Singapore). A total of 8 weevil samples originated from 8 populations—Cameroon (CAM), South Riau (SR), North Sarawak (NS), South Sarawak (SS), North Peninsular Malaysia (NPM), Central Peninsular Malaysia (CPM), South Peninsular Malaysia (SPM) and Sabah (SBH)—were genotyped to test for amplifiability and polymorphism of these 120 SSR markers. The PCR-based genotyping was carried out in 10 μL reactions consist of approximately 10 ng of genomic DNA, 7 µL 1x MyTaq Bioline buffer, 0.05 µM of forward and reverse primer, and 0.1 µM of fluorescence dye (FAM, HEX, NED or PET; Applied Biosystem, US) using Biometra PCR Thermocycler (Analytik Jena, Berlin, Germany). The PCR program was set at 95 °C for 60 s, 35 cycles of 95 °C for 15 s, and an annealing temperature of 54–58 °C for 15 s, 72 °C for 10 s, and 72 °C for 8 min. Genotypic data were generated in an ABI 3730 DNA Analyzer and Gene Mapper 7 program (Applied Biosystems, Massachusetts, USA). The amplicon of each polymorphic SSR was validated through single pass Sanger Sequencing using a genetic analyzer (Applied Biosystems, Massachusetts, USA). 

### 2.5. Genetic Diversity and Clustering Analysis

Only SNP and SSR markers with a call rate >95% were included in the genetic diversity analyses [32]. At the marker level, genetic diversity parameters, including major allele frequency (MAF), mean number of alleles (N_A_), expected heterozygosity (H_e_), observed heterozygosity (H_o_), polymorphism information content (PIC) [35], and inbreeding coefficient (F_is_) [36], were generated in the PowerMarker v3.25 program [37] for SSR and SNP, respectively. Same parameters of each marker system were also estimated at the population level, i.e., the assayed 12 weevil populations (Table 1). In the PowerMarker v3.25 program, genetic relatedness among 12 assayed weevil populations was subsequently estimated based on pairwise Nei’s genetic distance (D) [38] to construct a phenogram using the unweighted pair group method with arithmetic average (UPGMA) approach. These phenograms were visualized in the TreeView program [39]. In addition, principal component analyses (PCA) were performed on 180 individual weevils in R packages, namely adegenet [40], Hiefstat [41] and Pegas v 3.2.4 [42], following the references for Population Genetics in R [43]. The frequency of the null alleles (NAF) of SSR was further investigated in MICRO-CHECKER v2.2.3 [44] using the Brookfield 1 method for frequency estimation [45]. Allelic frequencies of SSR markers were corrected using the same program, followed by the re-estimation of SSR-based F_is_ across the populations using the PowerMarker v3.25 program [37]. 

## 3. Results

### 3.1. SNP Discovery and Marker Informativeness

**Figure 1:**
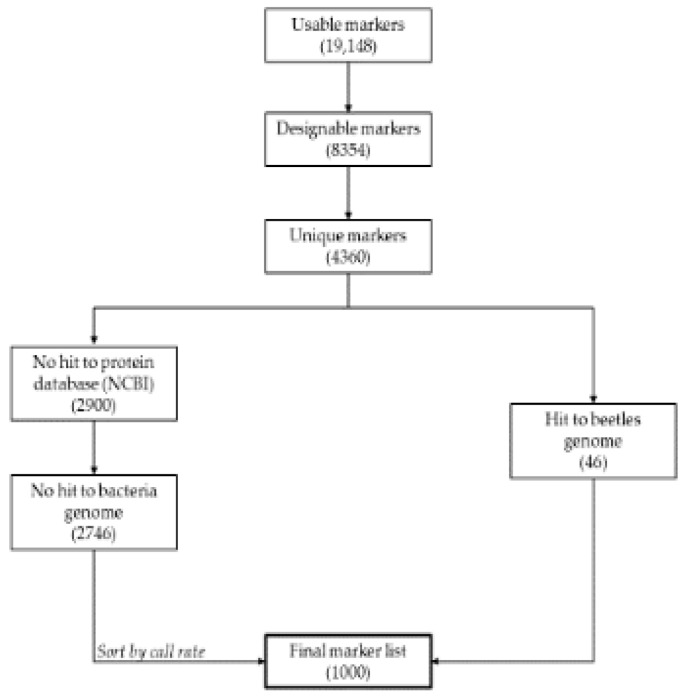
A flowchart of SNP filtration steps. The number in brackets at each filtration step represents the number of markers.

The RAD tag sequencing produced a total of 190,055,454 reads, with an average of 1.98 million reads per weevil, out of which 1,911,233 sequences were clustered and used for marker discovery. The number of SNPs obtained in each filtering step is shown in Figure 1. The initial number of SNPs discovered in 48 weevil samples was 19,148 SNP. For probe design, the number was reduced to 8354 SNP with the length of the flanking sequences of at least 75 bp upstream and downstream. Only 4360 SNP were found to be unique, and the number subsequently dropped to 2746 SNP that had no hit to the protein database and bacterial genome from the NCBI database. From this pool, as well as an additional 46 SNPs with good hits to the NR database, a total of 1000 SNP were eventually shortlisted and deposited in the European Variation Archive (EVA) database. Nevertheless, out of these 1000 SNPs with a mean call rate of 0.53% (±0.4322), only 220 SNPs with a call rate > 95% (Table S1) were selected for genetic diversity analyses. The informativeness of the selected 220 SNPs was first evaluated using mean PIC per SNP = 0.2387 (±0.1280), ranging from 0.0000 to 0.3750. Furthermore, the mean H_o_ per SNP was 0.2917 (±0.2221), which was close to the mean H_e_ per SNP = 0.2982 (±0.1742). In this study, N_A_ = 435 were detected, with the average of 1.9773 alleles per SNP (Table S1).

### 3.2. SSR Discovery and Marker Informativeness

A total of 223,200 SSR motifs were identified from the contigs, with an average length of 121 bp within the range between 103 bp and 320 bp. Only 645 SSR motifs were flanked with a sufficient length of sequences for primer designs. The number was further reduced to 120 sequences due to a good predicted match of primer pairs. The 120 designed primers were tested in a subset of 8 samples representing 1 native and 7 introduced populations for amplifiability and polymorphism tests. The results show that 30 SSR consistently produced amplicons with the expected size, with only 8 SSR found to be polymorphic (Table 2), while the remaining 22 monomorphic SSR are shown in Table S2. In addition, these polymorphic SSR, which were successfully validated based on single pass sequencing results (Supplementary Figures S1–S8), were subjected to full genotyping, with 180 weevil samples representing 12 populations, as shown in Table 1.

A total of 50 alleles were detected in the sample pool, and the mean N_A_ per SSR was 6.25 (±3.2) alleles, ranging from 4 to 13 alleles (Table 2). Moreover, the mean H_o_ per SSR = 0.2747 ±0.1614 was lower than the mean H_e_ per SSR = 0.5550 ±0.1934. The PIC per SSR was between the range of 0.2540 and 0.8024, with the mean of 0.5084 (±0.1928). All eight SSR markers indicated a significant deviation from the Hardy–Weinberg equilibrium (HWE) at *p* = 0.05 threshold (Table S3), but no strong linkage disequilibrium (LD) was detected among them (r^2^ < 0.5) (Table S4). 

Unfortunately, null alleles were detected in all polymorphic SSR, with an NAF (null allele frequency) ranging from 0.0341 to 0.3361 and the mean of 0.1903 (±0.1243) (Table 2). There are three classes of NAF: negligible (*NAF <* 0.05), moderate (0.05 ≤ NAF < 0.20), or large (NAF ≥ 0.20 [46]. Thus, two SSR, namely SDPek_R0022 (0.0341) and SDPek_R0142 (0.0390), exhibited low NAF, followed by SDPek_R0139 (0.0970) and SDPek_R0064 (0.1495), with moderate frequencies. The remaining four SSR showed a high incidence of null alleles (NAF > 0.2). 

### 3.3. Genetic Diversity Analysis

The genetic diversity of 12 assayed weevil populations was analyzed using 220 SNP and 8 SSR, respectively, to compare the applicability of both marker systems. For SNP, MAF of the CAM population (0.8222) and Ghana (GHN) population (0.9199) were higher than 0.7974 (±0.0409), which is the MAF mean (Table 3). This also resulted in a lower N_A_ mean (CAM = 1.6500 and GHN = 1.3318), H_o_ (CAM = 0.2417 and GHN = 0.0992), and PIC (CAM = 0.1810 and GHN = 0.0833) in both native populations, compared to the mean values across populations (mean N_A_ = 1.7690 ± 0.1535, H_o_ = 0.2914 ± 0.0649, and PIC = 0.2102 ± 0.0425). Interestingly, these four diversity parameters were comparable among the introduced populations and higher than the mean values across populations. 

In Table 3, using SNP, all populations recorded a negative F_is_, ranging from −0.1146 (Central West Kalimantan, CWK) to −0.0244 (CAM), except for the GHN population, with 0.0330. The SSR markers, however, indicated the opposite trend of F_is_ , in which all 12 populations showed positive values in the range between 0.2931 (South Sarawak, SS) to 0.5857 (North Peninsular Malaysia, NPM). As this was observed due to the presence of null alleles in some markers, allelic frequencies were then adjusted for re-estimation of SSR-based F_is_. After the adjustment, the SSR-based F_is_ were reduced, ranging from −0.0246 (CAM) to 0.1173 (SS). Particularly, the SBH (−0.0024), CWK (−0.0030), and CAM (−0.0246) populations recorded negative F_is_ values (Table 3). As expected, all assayed populations carried more SSR alleles (mean N_A_ ≥ 3.0000) than did the SNP, leading to a lower MAF mean (0.6370 ± 0.0665) (Table 3). However, the GHN population still retained the highest MAF, with 0.7542, and the lowest H_o_, with 0.1500. More alleles per SSR conferred better segregations of H_o_ and PIC across the populations. For instance, the CWK and SBH populations indicated the highest H_o_, with 0.3417, and the highest PIC, with 0.5142, respectively. 

### 3.4. Genetic Distance Estimate and Clustering Analysis

**Figure 2:**
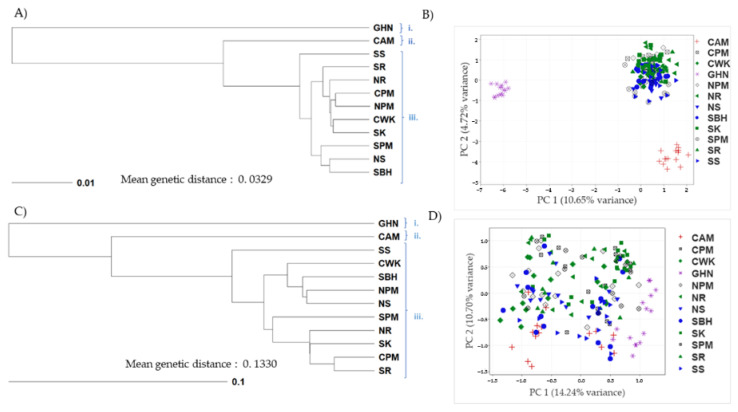
Phenogram and genetic clustering of 180 *E. kamerunicus* collected from 12 populations using (**A**,**B**) 220 SNP and (**C**,**D**) 8 SSR. Both SNP and SSR mutually assigned the assayed 12 populations into 3 major divisions (2 lineages and 1 cluster): (i) GHN, (ii) CAM, and (iii) introduced populations in Malaysia and Indonesia. GHN = Ghana, CAM = Cameroon, SS = South Sarawak, SR = South Riau, NR = North Riau, CPM = Central Peninsular Malaysia, NPM = North Peninsular Malaysia, CWK = Central West Kalimantan, SK = South Kalimantan, SPM = South Peninsular Malaysia, NS = North Sarawak, SBH = Sabah, East Malaysia.

In general, the mean pairwise Nei’s genetic distance (D) among the 12 assayed populations calculated using SNP and SSR was 0.0329 and 0.1330, respectively. The lowest genetic relatedness between the GHN population and the CAM population was revealed by SNP (D = 0.1452) and SSR (D = 0.3841). However, the closest related population pair detected in both marker systems varied: NPM-CPM pair (D = 0.011) in SNP and CPM-SR pair (D = 0.0367) in SSR. The genetic relatedness among populations was subsequently visualized in a UPGMA phenogram, based on D estimates (Figure 2). Both SNP and SSR mutually assigned the assayed 12 populations into 3 major divisions (2 lineages and 1 cluster): (i) GHN, (ii) CAM, and (iii) introduced populations in Malaysia and Indonesia (Figure 2A,C). Apparently, the introduced cluster was more genetically related to the native CAM than that of the native GHN. Besides similarity clustering, PCA plots (Figure 2B,D) further explained the resolution of both marker systems. The assayed SNP panel indicated a much higher resolution by tightly isolating the three clusters (Figure 2B), compared to assaying with the SSR panel (Figure 2D). The assayed 220 SNP provided a higher resolution than did the 8 SSR markers, with a reduced within-group variation and an increased between-group distance. 

## 4. Discussion

### 4.1. Nuclear DNA Marker Development and Informativeness

RAD tag sequencing is a cost-effective and rapid method for discovering DNA markers, especially for non-model species with no prior genome references [47,48,49,50,51]. The method enabled the identification of 19,148 SNP and 223,200 SSR motifs from 48 weevils. Normally, SNPs were expected to be more abundant throughout the genome of most species compared to SSR [52,53], but this was not observed in this study. The identification of SNP is dependent on the presence of nucleotide variants across individuals; therefore, overrepresentation of the introduced populations in Malaysia and Indonesia might limit the genetic polymorphism. Hence, the inclusion of a wider collection of native populations is recommended. For example, in the SNP discovery to study the population structure of the Asian longhorned beetle, the longhorned beetle samples from a wide coverage area in China and Korea were used [54]. 

Although discovery of SSR is more feasible, even with a single reference genome, only 0.28% of the identified SSR in this study were suitable for primer design. This was due to short generated RAD tag sequences, which usually spanned between 150 bp and 300 bp [55], thereby limiting the availability of flanking sequence for primer designs. A similar experience was reported in a previous study: only 237 out of 94,851 reads with SSR motif (0.24%) of an endangered yew species, *Taxus florinii*, were suitable for primer design [56]. In that study, the generated RAD tag sequences were on average 141 bp. Besides, in the absence of the *E. kamerunicus* reference genome, the identification of weevil-specific SNPs and SSRs, based on cross-mapping with reference genomes of the order Coleoptera, was found to be promising. A similar SNP marker discovery using RAD tag sequencing for a coconut leaf beetle without a reference genome was reported in [57]. As an example in plants, , the conserved DNA markers of the oil palm has successfully improved the genome assemblies in two other economically important palm species: the coconut and date palms [58].

Despite the reduced number of DNA markers, 220 SNP and 8 SSR were found to be informative. Markers with PIC values ≥0.5 are usually considered as highly informative, 0.25–0.5 as moderate, and <0.25 as low [35]. For bi-allelic SNP, the maximum PIC value per marker can only achieve 0.345 [59]. This explains why multi-allelic SSRs generally exhibited a higher PIC than that of the assayed SNP. The constrained informativeness of SNP, nevertheless, can always be compensated by using a large number of SNPs with high genotyping throughput. In this study, the N_A_ of SNP = 435 was observed to be 8.7 times higher than the N_A_ of SSR at 50.

### 4.2. Applications of Weevil-Specific SNP and SSR in Genetic Diversity Assessment

Both SNP and SSR mutually assigned 12 assayed populations into three major divisions: 2 lineages namely (i) GHN, (ii) CAM, and a cluster of (iii) introduced populations in Malaysia and Indonesia. With a higher mean N_A_ of multi-allelic SSR, the marker system had more advantages in explaining within-cluster variation than inter-clusters (Figure 2C,D). Previous studies reported that SSR was far more informative than SNP for population structure inference, where a randomly chosen set of SSRs would have 4–12 times greater informativeness than a random chosen set of SNPs [60,61]. For example, a total of 20 SSR markers were reported as useful for measuring gene heterozygosity and elucidating the population genetic structure of the adzuki bean weevil [62]. However, as mentioned earlier, SNP has a higher genotyping throughput compared to SSR, to compensate for the shortcoming. This feature enabled the assayed 220 SNP to confer better PCA clustering among the three groups, with reduced within-group variation and increased between-group distance. Similarly, a total of 239 SNP of red mangrove species effectively provided greater resolution on the population structure compared to the 8 SSR markers [63].

From the UPGMA phenogram, the cluster of the introduced populations in Malaysia and Indonesia was more closely related to CAM than GHN, which corresponded to the introduction event of the weevil from Cameroon to Southeast Asia. Furthermore, separate genetic clustering between Ghana, Central Africa, and Cameroon, West Africa, is in agreement with that obtained in a previous study reporting the difference in mitochondrial genetic clustering of *E. kamerunicus* between Central and West Africa [21]. 

Interestingly, 11 weevil populations indicated excessive SNP-based heterozygosity (F_is_ < 0), suggesting an absence of inbreeding in these populations. These findings might further support those of previous studies. Morphometric measurements of *E. kamerunicus* in Malaysia and Indonesia were not significantly different from those of the native Liberia weevils [64]. The population abundance of *E. kamerunicus* has also been reported to be stable [65]. It is worth noting that both SNP and SSR revealed unexpectedly poorer PIC and H_o_ in native GHN and CAM populations compared to the introduced populations, leading to a homozygosity deficit (F_is_ > 0) in the CAM population. One possible reason for this was non-random sampling, where these samples were collected from a limited number of palms, thereby not representing the heterozygosity of the real populations. Hence, random sampling from a wider range of the native population is recommended in future studies.

On the other hand, SSR-based F_is_ showed the total opposite results, with all populations recording positive values, suggesting excess homozygosity which can be partly due to the presence of null alleles [66]. Unfortunately, null alleles with high NAF > 0.2 were present in half of the assayed SSR (Table 2). Despite the reduction in the adjusted SSR-based F_is_, most values remained positive across the populations (Table 3). A null allele event, which was first reported in 1993, can occur when alleles fail to amplify due to the mutated annealing sites of SSR primers, leading to incorrect scoring of two different alleles as one allele [67]. This means that the genotyping of the samples may appear homozygous rather than heterozygous for the null allele. Certainly, underestimation of the heterozygosity parameter, such as Wright’s inbreeding estimates, can arise [68,69,70]. For instance, the estimated F_is_ of the oriental fruit moth populations derived from 10 SSR with high NAF was reported to be two to three times higher than the estimation derived from another set of 10 SSR with low NAF [71]. In this study, it is suggested that the existence of null allele in SSR has led to overestimation of homozygosity of assayed weevil populations, hence contributing to the positive values of SSR-based F_is_. Consequently, the generated positive F_is_ does not correctly represent inbreeding occurrence in both native and introduced *E. kamerunicus* populations. 

The presence of null alleles predicates the need to redesign the primers [70], but that was not feasible in this study. The length of the flanking region of RAD tag-SSR was extremely limited. Therefore, RAD tag-derived SNP was clearly more advantageous over RAD tag-derived SSR markers. The sequencing of the weevil genome as a reference is recommended to minimize the presence of null alleles, as illustrated in the Asian honeybee [72]. The SSR markers developed in this study can be deployed across other oil palm-pollinating weevil, especially from the *Elaeidobius* genus, i.e., *E. plagiatus, E. singularis, E. bilineatus, E. subvittatus*, and *E. spatilifer.* Closely related species would be expected to share more conserved sequence sites [73,74]. If the sequence is conserved between the model and the species of interest, the primers that flank the conserve regions will have a higher probability of amplifying DNA fragments in the species of interest [73]. Many successful cross-taxa applications of DNA markers, such as planthopper *Laodelphax striatellus*, with its related taxa [75], *Diabrotica* beetles [76], and oil palm *Elaeis guineensis*, with its close relative *E. oleifera* [77], were reported. 

These 220 informative SNP markers can be deployed as a PCR-based genotyping method, such as Seq-SNP (LGC Genomics, Berlin, Germany), Kompetitive Allele Specific PCR (KASP) (LGC Genomics, Hoddesdon, UK) [78] and TaqMan^®^ (Applied Biosystem, Massachusetts, USA) to obtain consistent and high call rates for more reliable results in future studies. In addition, PCR-based SNP markers can be cost-effective when genotyping a large number of samples. The Seq-SNP genotyping takes 3 days and costs USD 7 for each DNA sample genotyped with 163 SNPs in 261 cucumber varieties [25]. This feature is crucial for other researchers to extend similar surveys in different areas of interest and eventually, to form a comprehensive genetic diversity profile of weevil populations across Africa and Southeast Asia.

## 5. Conclusions

The discovery of nuclear DNA markers for *E. kamerunicus* using RAD tag sequencing was promising. The applicability of informative SNP in the genetic diversity assessment of weevil populations was observed to be superior to SSR. The main limitation of SSR is the null allele, which is common in RAD tag sequences. The presence of null allele in SSR markers from this study has led to an upward bias in inbreeding coefficient estimates. The inbreeding coefficient that was estimated using SNP-based data has recorded negative values in introduced *E. kamerunicus* populations indicating inbreeding is not being observed for now. For further confirmation, we propose expanding the survey to more populations across Malaysia, Indonesia, and Papua New Guinea using the developed specific nuclear DNA markers. Collaborative studies can be performed among industry players in oil palm-growing countries. This would obtain a more comprehensive genetic diversity concerning weevil populations in these regions. The information will be used to create guidelines regarding conservation planning and sustainability monitoring of this important pollinator species, from the estate to the national level. 

## Figures and Tables

**Table 1:** Localities of 12 assayed *E. kamerunicus* populations in Malaysia, Indonesia, Ghana, and Cameroon.

No.	Population	Pop. Code	Estate	Latitude	Longitude	Sample Size
**Native Population**						
1	Cameroon	CAM	Buea	5.2916532	9.4208524	15
2	Ghana	GHN	Kusi	6.1104472	−0.2673795	15
**Introduced population**						
3	South Riau, Indonesia	SR	Bhumireksa Nusasejati	0.1172028	103.6054083	15
4	North Riau, Indonesia	NR	Menggala	2.8770875	103.7287832	15
5	Central West Kalimantan, Indonesia	CWK	Sekunyir	−2.4329821	112.000172	15
6	South Kalimantan, Indonesia	SK	Angsana	3.6063617	115.5897206	15
7	North Peninsular Malaysia	NPM	Sungai Dingin	5.3678468	100.7063803	15
8	Central Peninsular Malaysia	CPM	West	3.4032204	101.4013073	15
9	South Peninsular Malaysia	SPM	Yong Peng	2.0160711	102.9960109	15
10	South Sarawak, East Malaysia	SS	Sessang	1.8433571	111.2101607	15
11	North Sarawak, East Malaysia	NS	Bayu	3.3757040	113.4109915	15
12	Sabah, East Malaysia	SBH	Tiger	4.4069250	117.8172309	15
					Total	180

**Table 2:** Eight polymorphic SSR and their marker characteristics.

No.	SSR Marker	Primer Sequence (5′-3′)	Repeat Motif	Size Range (bp)	Ta (°C)	MAF	N_A_	N_G_	PIC	NAF
1	SDPek_R0022	F: GCCTATAATAGACCGTTTGG; R: GTGAAGAACATTGTAACATCTC	(AAC)6	124–136	56	0.4306	6.00	15.00	0.6651	0.0341
2	SDPek_R0032	F: CGCTCTCCTCCTCATTATCA; R: TCTCTTGCATCTAGGTAACG	(AG)6	168–174	54	0.7306	5.00	8.00	0.3937	0.2875
3	SDPek_R0064	F:GTCACCAATAAGTTCCAAAGC; R: GTGGTGTTGGTGAACCTGAT	(CTGCA)4	185–200	56	0.8389	4.00	7.00	0.2540	0.1495
4	SDPek_R0079	F: TATTTGGATGTATTTCGGTTTG; R: GCGAGTATTTGTAGCGATC	(T)10	146–150	54	0.3939	9.00	17.00	0.6695	0.2875
5	SDPek_R0082	F: TCTCAAGGTGGCTCTCAT; R: CACATCTATCCGCACTACA	(T)13	117–130	56	0.2961	13.00	27.00	0.8024	0.2917
6	SDPek_R0139	F: CTCCAGTTATAGTACCACAATG; R: CGACTCGGCTCTTGTATT	(AG)6	131–139	54	0.5389	5.00	11.00	0.5401	0.0970
7	SDPek_R0142	F: CCTAAATAAGGACCACCCTA; R: CGACCTGTTAGCCTCTAC	(AT)5	132–140	54	0.5559	4.00	6.00	0.4294	0.0390
8	SDPek_R0147	F: TTGGTTCTATCGAGTAATGC; R: GCAATTTACCTCCAATGACA	(ATG)5	144–156	54	0.7944	4.00	8.00	0.3131	0.3361
				Mean		0.5724	6.25	12.38	0.5084	0.1903
				Standard deviation		0.1982	3.2	7.09	0.1928	0.1243

T_a_—annealing temperature, MAF—major allele frequency, PIC—polymorphism information content, N_A_—number of alleles, N_G_—number of genotypes, NAF—null allele frequency (null allele frequency is based on Brookfield 1 estimates [45].

**Table 3:** Genetic diversity parameters of 180 samples in 12 populations using 220 SNP and 8 SSR.

Pop. Code	N	SNP						SSR						
		MAF	Mean N_A_	H_e_	H_o_	PIC	F_is_	MAF	Mean N_A_	H_e_	H_o_	PIC	F_is_	Adjusted F_is_
SR	15	0.7849	1.8000	0.2795	0.3125	0.2226	−0.0837	0.6833	3.2500	0.4236	0.2500	0.3747	0.4381	0.0110
NS	15	0.7748	1.8409	0.2904	0.3181	0.2312	−0.0608	0.6000	3.5000	0.4878	0.3083	0.4454	0.3973	0.0501
CAM	15	0.8222	1.6500	0.2282	0.2417	0.1810	−0.0244	0.6542	3.0000	0.4497	0.2583	0.3945	0.4534	−0.0246
CWK	15	0.7764	1.8455	0.2908	0.3339	0.2316	−0.1146	0.5625	3.3750	0.5283	0.3417	0.4732	0.3831	−0.0030
GHN	15	0.9199	1.3318	0.1035	0.0992	0.0833	0.0330	0.7542	3.1250	0.3375	0.1500	0.3131	0.5789	0.1042
NR	15	0.7860	1.8364	0.2799	0.3073	0.2236	−0.0637	0.6417	3.2500	0.4508	0.2833	0.3931	0.4009	0.1470
NPM	15	0.7804	1.8409	0.2886	0.3222	0.2306	−0.0822	0.5500	3.8750	0.5525	0.2417	0.5037	0.5857	0.0667
SK	15	0.7759	1.8364	0.2908	0.3179	0.2313	−0.0588	0.6625	3.5000	0.4431	0.3083	0.4049	0.3350	0.1154
SS	15	0.7947	1.7955	0.2684	0.3031	0.2147	−0.0955	0.6423	3.3750	0.4551	0.3363	0.3988	0.2931	0.1173
SBH	15	0.7845	1.8409	0.2803	0.3055	0.2241	−0.0553	0.5250	3.8750	0.5650	0.2583	0.5142	0.5667	−0.0024
CPM	15	0.7742	1.8409	0.2950	0.3319	0.2352	−0.0911	0.6958	3.3750	0.4242	0.2583	0.3808	0.4198	0.0392
SPM	15	0.7947	1.7864	0.2673	0.3034	0.2131	−0.1008	0.6723	3.1250	0.4308	0.3036	0.3812	0.3269	0.0623
Mean		0.7974	1.7690	0.2636	0.2914	0.2102	−0.0665	0.6370	3.3854	0.4624	0.2748	0.4148	0.4316	0.0569
Standard Dev		0.0409	0.1535	0.0535	0.0649	0.0425	0.0397	0.0665	0.2742	0.0632	0.0518	0.0585	0.0992	0.0554

MAF—major allele frequency, N—number of analyzed individual, Mean N_A_ = mean number of allele, H_e_—expected heterozygosity, H_o_—observed heterozygosity, PIC—polymorphism information content, F_is_—fixation index in the population, adjusted F_is_—adjusted fixation index in the population.

## Data Availability

The datasets generated for this study can be found in the European Nucleotide Archive (ENA) with Accession No. GCA_945836925. A list of selected SNP markers has been deposited in the European Variation Archive (EVA), with Accession No. PRJEB55362.

## Supplementary Materials

The following supporting information can be downloaded at: https://www.mdpi.com/article/10.3390/insects14020157/s1, Figures S1–S8: Validation of 8 polymorphic SSR markers through Sanger sequencing; Table S1: List of 220 SNP markers with primer sequences; Table S2: List of 22 monomorphic markers with primer sequences; Table S3: Hardy–Weinberg Equilibrium (HWE) of 8 SSR markers; Table S4: Pairwise calculation of linkage disequilibrium (LD) for 8 SSR markers.

## References

[1] Pamin K.A. A hundred and fifty years of oil palm in Indonesia: From the Bogor Botanical Garden to the industry. Proceedings of the International Oil Palm Conference ‘Commodity of the Past, Today and the Future’.

[2] Corley R.H., Tinker P. (2003). Chapter 1. The Origin and Development of the Oil Palm Industry. The Oil Palm.

[3] Parveez G.K.A. (2021). Oil palm economic performance in Malaysia and R&D progress in 2020. J. Oil Palm Res..

[4] Syed R.A. (1979). Studies on oil palm pollination by insects. Bull. Entomol. Res..

[5] Hardon J., Wastie R.L., Earp D.A. (1973). Assisted pollination in oil palm: A review. Advances in Oil Palm Cultivation.

[6] Wahid M.B. (1984). Developments of the Oil Palm Pollinator, *Elaiedobius kamerunicus* in Malaysia. Palm Oil Dev..

[7] Syed R. (1982). Insect Pollination of Oil Palm: Feasibility of Introducing Elaeidobius Spp. into Malaysia. Oil Palm in the Eighties. A Report of the Proceedings of the International Conference on Oil Palm in the Eighties.

[8] Kang S., Zam A. Quarantine aspects of the introduction into Malaysia of an oil palm insect pollinator. Proceedings of the International Conference on Plant Protection in the Tropics.

[9] Tuo Y., Koua H.K., Hala N. (2011). Biology of *Elaeidobius kamerunicus* and *Elaeidobius plagiatus* (Coleoptera: Curculionidae) main pollinators of oil palm in West Africa. Eur. J. Sci. Res..

[10] Siswanto, Soetopo D. (2020). Population of oil palm pollinator insect (*Elaeidobius kamerunicus* Faust.) at PTP Nusantara VIII Cisalak Baru, Rangkasbitung-Banten. IOP Conf. Ser. Earth Environ. Sci..

[11] Corley R.H.V., Tinker P.B. (2015). The Oil Palm.

[12] Syed R.A., Law I.H., Corley R.H. (1982). Insect pollination of oil palm: Introduction, establishment and pollinating efficiency of *Elaeidobius kamerunicus* in Malaysia. Planter.

[13] Mariau D., Genty P. (1988). IRHO contribution to the study of oil palm insect pollinators in Africa, South America and Indonesia. Oléagineux.

[14] Moslim R., Kamarudin N. (2016). Comparative Traits of Pollinating Weevils and Factors Affecting Its Population in Malaysia. Task Force Oil Palm Pollinating Weevil and Fruit Set.

[15] Donough C., Chew K., Law I. (1996). Effect of fruit set on OER and KER-results from studies at pamol estates. Plant.

[16] Rao V., Law I. (1998). The problem of poor fruitset in parts of East Malaysia. Plant.

[17] Ming K.S. (1999). The *Elaeidobius kamerunicus* story. Plant.

[18] Krantz G.W., Poinar G.O. (2004). Mites, nematodes and the multimillion dollar weevil. J. Nat. Hist..

[19] Nasir D.M., Mamat N.-S., Abdul Muneim N.A., Ong-Abdullah M., Abd Latip N.F.B., Su S., Hazmi I.R.B. (2020). Morphometric Analysis of the Oil Palm Pollinating Weevil, *Elaeidobius kamerunicus* (Faust, 1878) (Coleoptera: Curculionidae) from Oil Palm Plantations in Malaysia. J. Entomol. Res. Soc..

[20] Sukhodolskaya R. (2013). Intraspecific Body Size Variation in Ground Beetles (Coleoptera, Carabidae) in Urban-Suburban-Rural-Natural Gradient. Acta Biol. Univ. Daugavp.

[21] Haran J., Ndzana Abanda R.F.X., Benoit L., Bakoumé C., Beaudoin-Ollivier L. (2020). Multilocus phylogeography of the world populations of *Elaeidobius kamerunicus* (Coleoptera, Curculionidae), pollinator of the palm *Elaeis guineensis*. Bull. Entomol. Res..

[22] Ballard J.W.O., Whitlock M.C. (2004). The incomplete natural history of mitochondria. Mol. Ecol..

[23] Qin Y., Buahom N., Krosch M.N., Du Y., Wu Y., Malacrida A.R. (2016). Genetic diversity and population structure in *Bactrocera correcta* (Diptera: Tephritidae) inferred from mtDNA cox1 and microsatellite markers. Sci. Rep..

[24] Yang X., Xu Y., Shah T., Li H., Han Z., Li J., Yan J. (2011). Comparison of SSRs and SNPs in assessment of genetic relatedness in maize. Genetica.

[25] Zhang J., Yang J., Zhang L., Luo J., Zhao H., Zhang J., Wen C. (2020). A new SNP genotyping technology Target SNP-seq and its application in genetic analysis of cucumber varieties. Sci. Rep..

[26] Li W., Godzik A. (2006). Cd-hit: A fast program for clustering and comparing large sets of protein or nucleotide sequences. Bioinformatics.

[27] Li H., Durbin R. (2009). Fast and accurate short read alignment with Burrows-Wheeler transform. Bioinformatics.

[28] Garrison E.P., Marth G.T. (2012). Haplotype-based variant detection from short-read sequencing. arXiv.

[29] (2004). The NCBI C++ Toolkit.

[30] Altschul S.F., Gish W., Miller W., Myers E.W., Lipman D.J. (1990). Basic local alignment search tool. J. Mol. Biol..

[31] Purcell S., Neale B., Todd-Brown K., Thomas L., Ferreira M.A.R., Bender D., Maller J., Sklar P., de Bakker P.I.W., Daly M.J. (2007). PLINK: A tool set for whole-genome association and population-based linkage analyses. Am. J. Hum. Genet..

[32] Melville J., Haines M.L., Boysen K., Hodkinson L., Kilian A., Smith Date K.L., Potvin D.A., Parris K.M. (2017). Identifying hybridization and admixture using SNPs: Application of the DArTseq platform in phylogeographic research on vertebrates. R. Soc. Open Sci..

[33] Metz S., Cabrera J.M., Rueda E., Giri F., Amavet P. (2016). FullSSR: Microsatellite Finder and Primer Designer. Adv. Bioinform..

[34] Schuelke M. (2000). An economic method for the fluorescent labeling of PCR fragments A poor man ’ s approach to genotyping for research and high-throughput diagnostics. Nat. Biotechnol..

[35] Botstein D., White R.L., Skolnick M., Davis R.W. (1980). Construction of a genetic linkage map in man using restriction fragment length polymorphisms. Am. J. Hum. Genet..

[36] Wright S. (1965). The interpretation of population structure by F-statistics with special regard to systems of mating. Evolution.

[37] Liu K., Muse S.V. (2005). PowerMarker: An integrated analysis environment for genetic marker analysis. Bioinform. Appl. Notes.

[38] Nei M., Tajima F., Tateno Y. (1983). Accuracy of estimated phylogenetic trees from molecular data. J. Mol. Evol..

[39] Page R.D.M. (1996). Tree View: An application to display phylogenetic trees on personal computers. Bioinformatics.

[40] Jombart T. (2008). adegenet: A R package for the multivariate analysis of genetic markers. Bioinformatics.

[41] Goudet J. (2005). HIERFSTAT, a package for R to compute and test hierarchical F-statistics. Mol. Ecol. Notes.

[42] Paradis E. (2010). Pegas: An R Package for Population Genetics with an Integrated-Modular Approach. Bioinformatics.

[43] Kamvar Z.N., López-Uribe M.M., Coughlan S., Grünwald N.J., Lapp H., Manel S. (2017). Developing educational resources for population genetics in R: An open and collaborative approach. Mol. Ecol. Resour..

[44] Van Oosterhout C., Hutchinson W.F., Wills D.P.M., Shipley P. (2004). MICRO-CHECKER: Software for identifying and correcting genotyping errors in microsatellite data. Mol. Ecol. Notes.

[45] Brookfield J.F. (1996). A simple new method for estimating null allele frequency from heterozygote deficiency. Mol. Ecol..

[46] Chapuis M.-P., Estoup A. (2007). Microsatellite Null Alleles and Estimation of Population Differentiation. Mol. Biol. Evol..

[47] Peterson B.K., Weber J.N., Kay E.H., Fisher H.S., Hoekstra H.E. (2012). Double Digest RADseq: An Inexpensive Method for *De Novo* SNP Discovery and Genotyping in Model and Non-Model Species. PLoS ONE.

[48] Miller M.R., Dunham J.P., Amores A., Cresko W.A., Johnson E.A. (2007). Rapid and cost-effective polymorphism identification and genotyping using restriction site associated DNA (RAD) markers. Genome Res..

[49] Baird N.A., Etter P.D., Atwood T.S., Currey M.C., Shiver A.L., Lewis Z.A., Selker E.U., Cresko W.A., Johnson E.A. (2008). Rapid SNP discovery and genetic mapping using sequenced RAD markers. PLoS ONE.

[50] Ogden R., Gharbi K., Mugue N., Martinsohn J., Senn H., Davey J.W., Pourkazemi M., McEwing R., Eland C., Vidotto M. (2013). Sturgeon conservation genomics: SNP discovery and validation using RAD sequencing. Mol. Ecol..

[51] Pavinato V.A.C., Margarido G.R.A., Wijeratne A.J., Wijeratne S., Meulia T., Souza A.P., Michel A.P., Zucchi M.I. (2017). Restriction site associated DNA (RAD) for de novo sequencing and marker discovery in sugarcane borer, Diatraea saccharalis Fab. (Lepidoptera: Crambidae). Mol. Ecol. Resour..

[52] Guichoux E., Lagache L., Wagner S., Chaumeil P., Léger P., Lepais O., Lepoittevin C., Malausa T., Revardel E., Salin F. (2011). Current trends in microsatellite genotyping. Mol. Ecol. Resour..

[53] Chen W., Hou L., Zhang Z., Pang X., Li Y. (2017). Genetic Diversity, Population Structure, and Linkage Disequilibrium of a Core Collection of *Ziziphus jujuba* Assessed with Genome-wide SNPs Developed by Genotyping-by-sequencing and SSR Markers. Front. Plant Sci..

[54] Cui M., Wu Y., Javal M., Giguère I., Roux G., Andres J.A., Keena M., Shi J., Wang B., Braswell E. (2022). Genome-scale phylogeography resolves the native population structure of the Asian longhorned beetle, *Anoplophora glabripennis* (Motschulsky). Evol. Appl..

[55] Andrews K.R., Good J.M., Miller M.R., Luikart G., Paul A., Sciences W., Avenue O.S., Station L.B., Group W.G., Studies E. (2016). Harnessing the power of RADseq for ecological and evolutionary genomics. Nat. Rev. Genet..

[56] Qin H., Yang G., Provan J., Liu J., Gao L. (2017). Using MiddRAD-seq data to develop polymorphic microsatellite markers for an endangered yew species. Plant Divers..

[57] Chen Z., Wang G., Li M., Peng Z., Ali H., Xu L., Gurr G.M., Hou Y. (2020). Development of Single Nucleotide Polymorphism (SNP) Markers for Analysis of Population Structure and Invasion Pathway in the Coconut Leaf Beetle *Brontispa longissima* (Gestro) Using Restriction Site-Associated DNA (RAD) Genotyping in Southern China. Insects.

[58] Ong A.-L., Teh C.-K., Mayes S., Massawe F., Appleton D.R., Kulaveerasingam H. (2020). An Improved Oil Palm Genome Assembly as a Valuable Resource for Crop Improvement and Comparative Genomics in the Arecoideae Subfamily. Plants.

[59] Hildebrand C.E., Torney D.C., Wagner R.P. (1992). Informativeness of Polymorphic DNA Markers. Los Alamos Sci..

[60] Rosenberg N.A., Li L.M., Ward R., Pritchard J.K. (2003). Informativeness of genetic markers for inference of ancestry. Am. J. Hum. Genet..

[61] Liu N., Chen L., Wang S., Oh C., Zhao H. (2005). Comparison of single-nucleotide polymorphisms and microsatellites in inference of population structure. BMC Genet..

[62] Duan C., Li D., Sun S., Wang X., Zhu Z. (2014). Rapid Development of Microsatellite Markers for *Callosobruchus chinensis* Using Illumina Paired-End Sequencing. PLoS ONE.

[63] Hodel R.G.J., Chen S., Payton A.C., McDaniel S.F., Soltis P., Soltis D.E. (2017). Adding loci improves phylogeographic resolution in red mangroves despite increased missing data: Comparing microsatellites and RAD-Seq and investigating loci filtering. Sci. Rep..

[64] Latip N.F.A., Ghani I.A., Hazmi I.R., Cik Mohd Rizuan Zainal Abidin D.S. (2019). Morphometric Comparison of the Oil Palm Pollinator *Elaeidobius kamerunicus* Faust (Coleoptera: Curculionidae) from Malaysia, Indonesia, and Liberia. Coleopt. Bull..

[65] Nurul Fatihah A.L., Muhamad Fahmi M.H., Luqman H.A., Syarifah Nadiah S.M.D., Teo T.M., Izfa Riza H., Idris A.B. (2019). Effects of Rainfall, Number of Male Inflorescences and Spikelets on the Population Abundance of *Elaeidobius kamerunicus* (Coleoptera: Curculionidae). Sains Malays..

[66] Pompanon F., Bonin A., Bellemain E., Taberlet P. (2005). Genotyping errors: Causes, consequences and solutions. Nat. Rev. Genet..

[67] Callen D.F., Thompson A.D., Shen Y., Phillips H.A., Richards R.I., Mulley J.C., Sutherland G.R. (1993). Incidence and origin of “null” alleles in the (AC)n microsatellite markers. Am. J. Hum. Genet..

[68] Chybicki I.J., Oleksa A., Burczyk J. (2011). Increased inbreeding and strong kinship structure in *Taxus baccata* estimated from both AFLP and SSR data. Heredity.

[69] Kelly A.C., Mateus-Pinilla N.E., Douglas M., Douglas M., Shelton P., Novakofski J. (2011). Microsatellites behaving badly: Empirical evaluation of genotyping errors and subsequent impacts on population studies. Genet. Mol. Res..

[70] Wu X., Wang L., Zhang D., Wen Y. (2019). Microsatellite null alleles affected population genetic analyses: A case study of Maire yew (*Taxus chinensis* var. *mairei*). J. For. Res..

[71] Song W., Cao L.-J., Wang Y.-Z., Li B.-Y., Wei S.-J. (2017). Novel microsatellite markers for the oriental fruit moth *Grapholita molesta* (Lepidoptera: Tortricidae) and effects of null alleles on population genetics analyses. Bull. Entomol. Res..

[72] Liu L., Qin M., Yang L., Song Z., Luo L., Bao H., Ma Z., Zhou Z., Xu J. (2017). A genome-wide analysis of simple sequence repeats in *Apis cerana* and its development as polymorphism markers. Gene.

[73] Wang M., Barkley N., Jenkins T. (2009). Microsatellite Markers in Plants and Insects. Part I: Applications of Biotechnology. Genes Genomes Genom..

[74] Ding S., Wang S., He K., Jiang M., Li F. (2017). Large-scale analysis reveals that the genome features of simple sequence repeats are generally conserved at the family level in insects. BMC Genom..

[75] Liu Y.-D., Zhang B., Hou M. (2014). Thirteen microsatellite loci for *Laodelphax striatellus* and cross amplification in related taxa. Entomol. Sci..

[76] Waits E.R., Stolz U.W.E. (2008). Polymorphic microsatellite loci from northern and Mexican corn rootworms (Insecta: Coleoptera: Chrysomelidae) and cross-amplification with other *Diabrotica* spp.. Mol. Ecol. Resour..

[77] Billotte N., Risterucci A.M., Barcelos E., Noyer J.L., Amblard P., Baurens F.C. (2001). Development, characterisation, and across-taxa utility of oil palm (*Elaeis guineensis* Jacq.) microsatellite markers. Genome.

[78] Alvarez-Fernandez A., Bernal M.J., Fradejas I., Martin Ramírez A., Md Yusuf N.A., Lanza M., Hisam S., Pérez de Ayala A., Rubio J.M. (2021). KASP: A genotyping method to rapid identification of resistance in *Plasmodium falciparum*. Malar. J..

