# Supplementary material for: Development of Nuclear DNA Markers for Applications in Genetic Diversity Study of Oil Palm-Pollinating Weevil Populations
Source: Insects. 2023 Feb 3;14(2):157. doi: 10.3390/insects14020157 (PMC9967927; doi:10.3390/insects14020157)
Supplement: Supplementary file 1 [file insects-14-00157-s001.zip › insects-2056860-supplementary/insects-2056860-supplementary/insects-2056860- Supplementary/Figures S1-8.pdf]

Supplementary Material

1 Supplementary Figures

Confirmation of SSR markers prime at targeted SSR by single pass sequencing of PCR amplicon. Sequences with SSR region, SSR pattern in box, with forward and reverse primers highlighted. Query is the sequence retrieved from RAD-tag sequencing; subject is the sequence retrieved from PCR-based product of the particular SSR genotyping in *E. kamerunicus*.

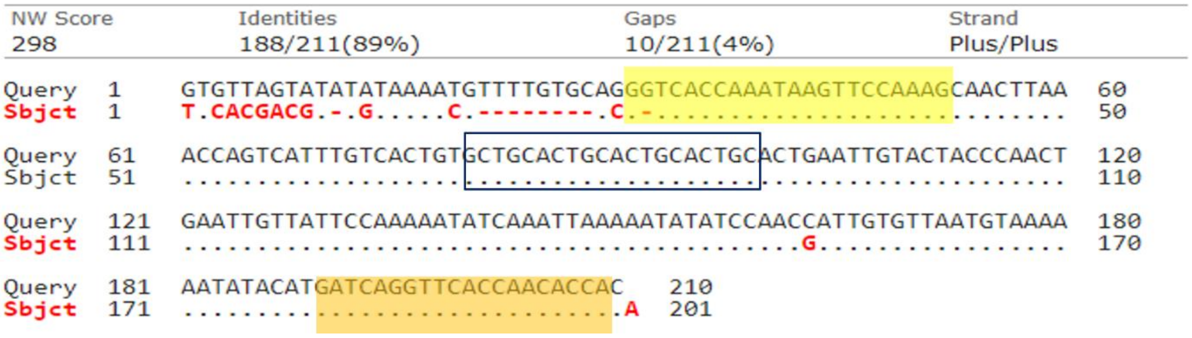

Figure S1. BLAST result of RAD tag sequence MC01881978 for SSR SDPek_R0064: Score 56.3, 18% query cover, E value 0.001, 92.11 to PREDICTED: alkali bee, *Nomia melanderi* ubiquitin carboxyl-terminal hydrolase 7-like (LOC116433232), mRNA Accession number: XM_031991116.1

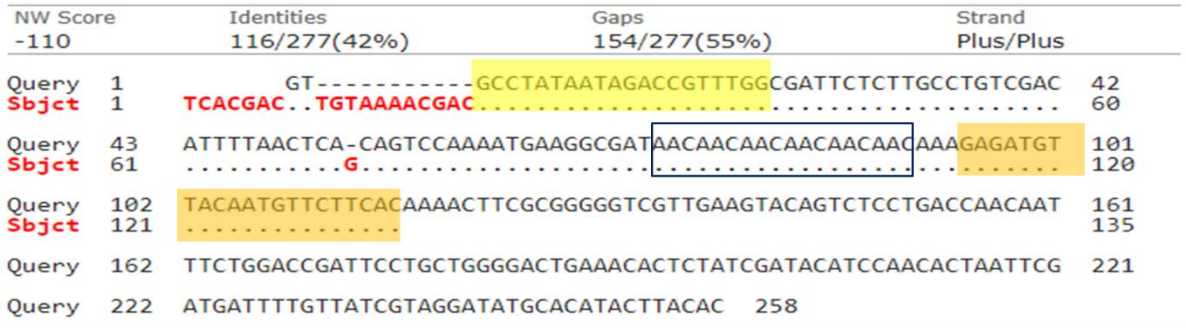

Figure S2. BLAST result of RAD tag sequence MC01272000 for SSR SDPek_R0022**.** Score 131, 47% query cover, E-value 2e-26, 83.61% identity to PREDICTED: Asian long-horned beetle (*Anoplophora glabripennis*) zinc finger protein 395 (LOC108904982), mRNA Accession number: XM_018707724.1

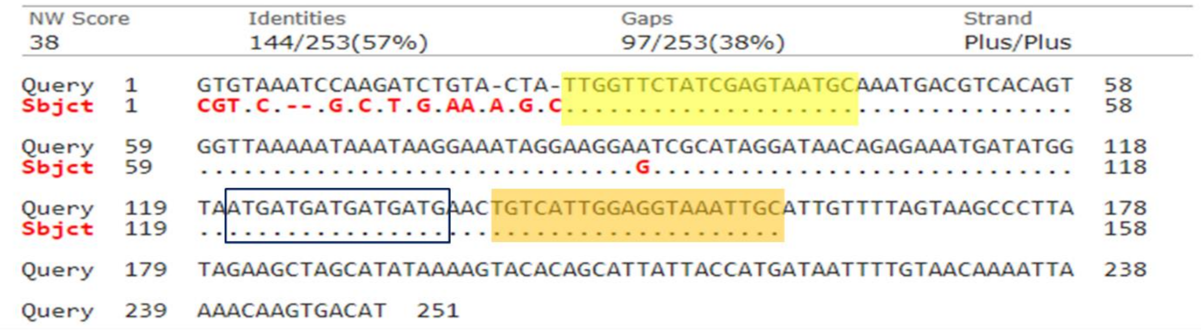

Figure S3. BLAST result of RAD tag sequence MC01269204 for SSR SDPek_R0147: Score 47.3, 11% query cover, E-value 0.63, identity 96.43% to ringlet butterfly, *Aphantopus hyperantus* genome assembly, chromosome: 21 Accession number: LR761668.1

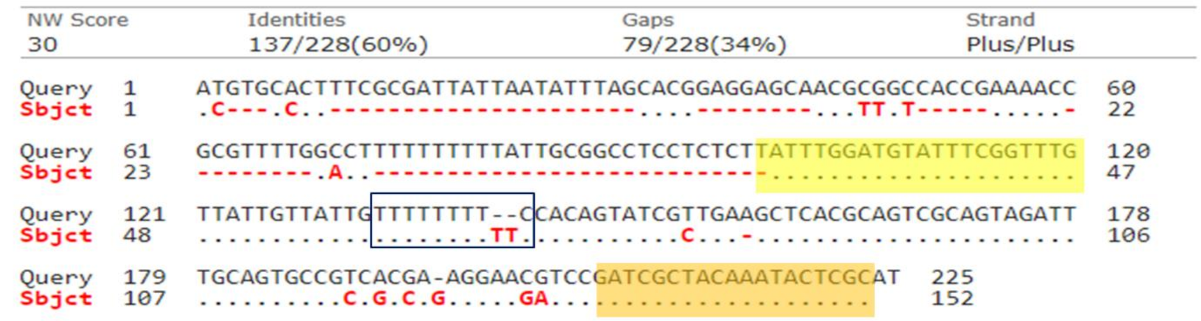

Figure S4. BLAST result of RAD tag sequence MC00810113 for SSR SDPek_R0079: Score 55.4, 13% query cover, E-value 0.001, identity 100.00% to PREDICTED: fruit fly, *Drosophila ananassae* glutathione hydrolase 1 proenzyme (LOC6505070), transcript variant X2, mRNA Accession number: XM_001965482.3

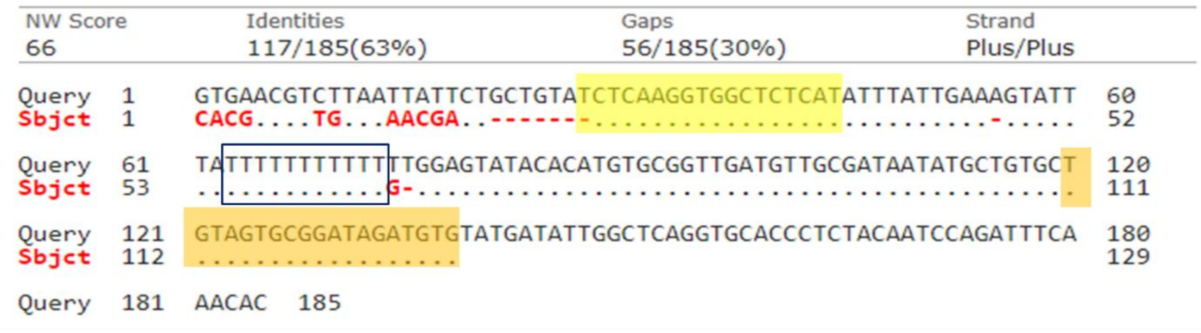

Figure S5. BLAST result: RAD tag sequence MC01709667 for SSR SDPek_R0082: Score 50.9, 18% query cover, E-value 0.036, identity 91.43% to *Drosophila melanogaster* strain rover (forR) chromosome X, Accession number: CP023335.1

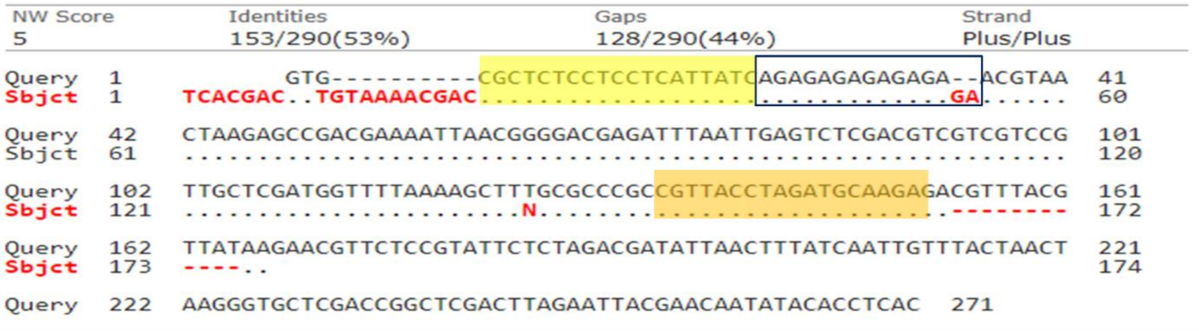

Figure S6. BLAST result for RAD tag sequence MC01833352 for SSR SDPek_R0032: Score 59.0, 32% query cover, E-value 1e-04, identity 76.09% to flightless weevil, *Eurhoptus pyriformis* isolate SSM555 internal transcribed spacer 1 and 5.8S ribosomal RNA gene, partial sequence Accession number: MH023494.1

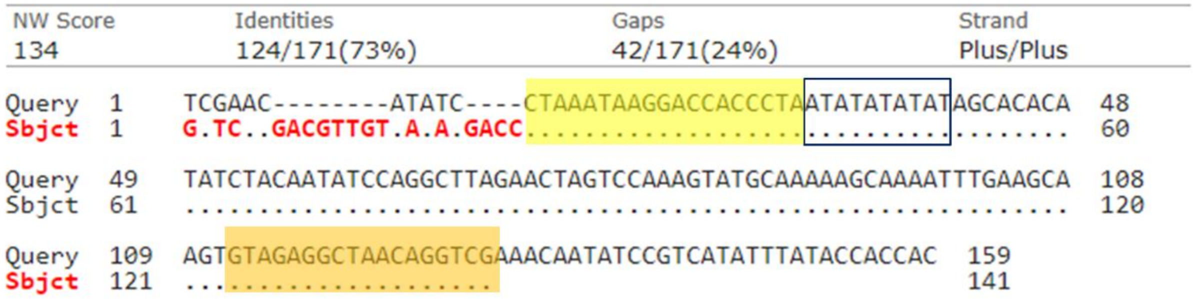

Figure S7. BLAST result for RAD tag sequence MC00712254 for SSR SDPek_R0142: Score 74.3, 28% query cover, E-value 5e-09, identity 81.82% to PREDICTED: turnip sawfly, *Athalia rosae* uncharacterized LOC105691158 (LOC105691158), transcript variant X2, mRNA Accession number: XM_020855342.2

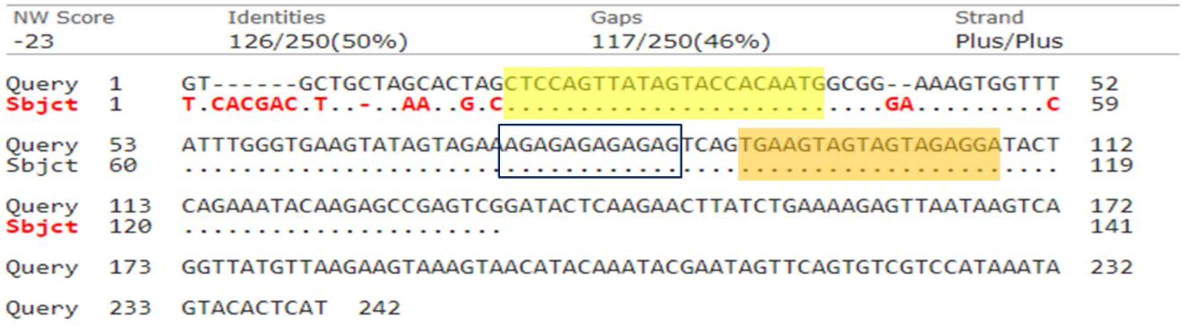

Figure S8. BLAST result for RAD tag sequence for SSR SDPek_R0139: Score 47.3, 17% query cover, E-value 0.61, identity 85.71% to PREDICTED: parasitoid, *Trichogramma pretiosum* alpha-tubulin N-acetyltransferase-like (LOC106652111), mRNA Accession number: XM_014370893.2
